# Natural and redesigned wasp venom peptides with selective antitumoral activity

**DOI:** 10.3762/bjoc.14.144

**Published:** 2018-07-06

**Authors:** Marcelo D T Torres, Gislaine P Andrade, Roseli H Sato, Cibele N Pedron, Tania M Manieri, Giselle Cerchiaro, Anderson O Ribeiro, Cesar de la Fuente-Nunez, Vani X Oliveira

**Affiliations:** 1 Centro de Ciências Naturais e Humanas, Universidade Federal do ABC, Santo André, 09210580, SP, Brazil; 2 Synthetic Biology Group, MIT Synthetic Biology Center, Research Laboratory of Electronics, Department of Biological Engineering, and Department of Electrical Engineering and Computer Science, Massachusetts Institute of Technology; Broad Institute of MIT and Harvard, The Center for Microbiome Informatics and Therapeutics, Cambridge, 02139, MA, United States of America

**Keywords:** breast cancer, decoralin, MCF-7 cells, peptide design, selective anticancer peptides, structure–activity relationships

## Abstract

About 1 in 8 U.S. women (≈12%) will develop invasive breast cancer over the course of their lifetime. Surgery, chemotherapy, radiotherapy, and hormone manipulation constitute the major treatment options for breast cancer. Here, we show that both a natural antimicrobial peptide (AMP) derived from wasp venom (decoralin, Dec-NH_2_), and its synthetic variants generated via peptide design, display potent activity against cancer cells. We tested the derivatives at increasing doses and observed anticancer activity at concentrations as low as 12.5 μmol L^−1^ for the selective targeting of MCF-7 breast cancer cells. Flow cytometry assays further revealed that treatment with wild-type (WT) peptide Dec-NH_2_ led to necrosis of MCF-7 cells. Additional atomic force microscopy (AFM) measurements indicated that the roughness of cancer cell membranes increased significantly when treated with lead peptides compared to controls. Biophysical features such as helicity, hydrophobicity, and net positive charge were identified to play an important role in the anticancer activity of the peptides. Indeed, abrupt changes in peptide hydrophobicity and conformational propensity led to peptide inactivation, whereas increasing the net positive charge of peptides enhanced their activity. We present peptide templates with selective activity towards breast cancer cells that leave normal cells unaffected. These templates represent excellent scaffolds for the design of selective anticancer peptide therapeutics.

## Introduction

Approximately 12% of U.S. women develop breast cancer according to the U.S. Breast Cancer website (http://www.breastcancer.org/symptoms/understand_bc/statistics). The current treatment approaches, which include surgery, chemotherapy, radiotherapy, and hormone manipulation, are highly invasive and present numerous deleterious side effects. Therefore, alternative anticancer therapies are needed both to destroy cancer cells and to avoid toxicity towards normal host cells.

Antimicrobial peptides (AMPs) are produced by the innate immune system of virtually every organism on Earth. These agents represent promising anticancer candidates since, in addition to their activity vs bacteria [1], viruses, parasites [2–8], and fungi [1,9–10], they can kill cancer cells [11]. So far, >2,500 AMPs have been described in the literature and only ≈10% of those are known to exhibit anticancer activity, according to the Antimicrobial Peptide Database (http://aps.unmc.edu/AP/main.php). In total, there are around 600 anticancer/antitumoral peptides according to the Database of Anticancer Peptides and Proteins (http://crdd.osdd.net/raghava/cancerppd/). Those AMPs with anticancer activity have been termed anticancer peptides (ACPs). Since their initial discovery, ACPs have constituted a promising alternative to conventional chemotherapy [11–12]. ACPs are promising anticancer compounds as they offer advantages such as higher specificity and lower incidence of acquired resistance in comparison to existing therapies [12–14].

ACPs derive from various sources and consequently share low homology [15–18]. These peptides have similar characteristics such as a positive charge, amphipathic structure, defined secondary structures in hydrophobic environments, and rapid anticancer activity [12,19]. Helical structures are the most common structural motifs of ACPs. Their stable amphipathic structures tend to be key for their anticancer activity, as they enable membrane binding [20]. Their anticancer activity typically occurs at micromolar concentrations [21] and is not usually accompanied by hemolytic activity probably because there are structural differences between the membranes of red blood cells and cancer cells, which are zwitterionic and negatively charged, respectively. Structure–activity relationship studies have identified amphiphilicity and polar angle as the most important physicochemical properties required for ACPs to invade cancer cells or disturb their membranes [22–23].

In 2007, Konno et al. described decoralin (Dec-Ser-Leu-Leu-Ser-Leu-Ile-Arg-Lys-Leu-Ile-Thr), an α-helical AMP from *Oreumenes decoratus* wasp venom [24]. In addition, the authors described its amidated analog (Dec-NH_2_), which displayed higher activity than its parent molecule against Gram-positive bacteria, Gram-negative bacteria, fungi, and protozoa. However, both peptides presented high hemolytic activity, which limited their use as potential therapies.

Torres et al. synthesized Dec-NH_2_ analogs with single and double substitutions, which exhibited increased resistance to degradation and lower hemolytic activity [9–10]. The two Dec-NH_2_ analogs designed to fit a leucine zipper (LZ) template [25–26] presented the lowest hemolytic activity against red blood cells and maintained the antimicrobial activity of the parent template molecule vs Gram-positive bacteria, Gram-negative bacteria, and fungi. The authors attributed these activities to the helical propensity of the designer peptides [9]. Another study further reengineered Dec-NH_2_ to generate seven analogs containing single or double substitutions [10]. These derivatives were designed to preserve specific physicochemical features, such as net positive charge, hydrophobicity, and amphipathicity, which are known to be important for interacting with membranes, exerting bioactivity against microorganisms and cancer cells, and suppressing unwanted hemolytic activity [10].

Since the aforementioned peptides were designed to target negatively charged bacterial membranes, we reasoned that their activity would translate to cancer cells, whose membranes also possess a net negative charge. We hypothesized that their conformational tendency and physicochemical properties would enable interactions with tumor cell membranes, leading to subsequent death. In the present study, we investigated Dec-NH_2_, its LZ template and single/double substituted derivatives for their ability to selectively kill MCF-7 breast cancer cells.

## Results and Discussion

### Peptide design, chemical synthesis, purification and physicochemical analyses

**Figure 1:**
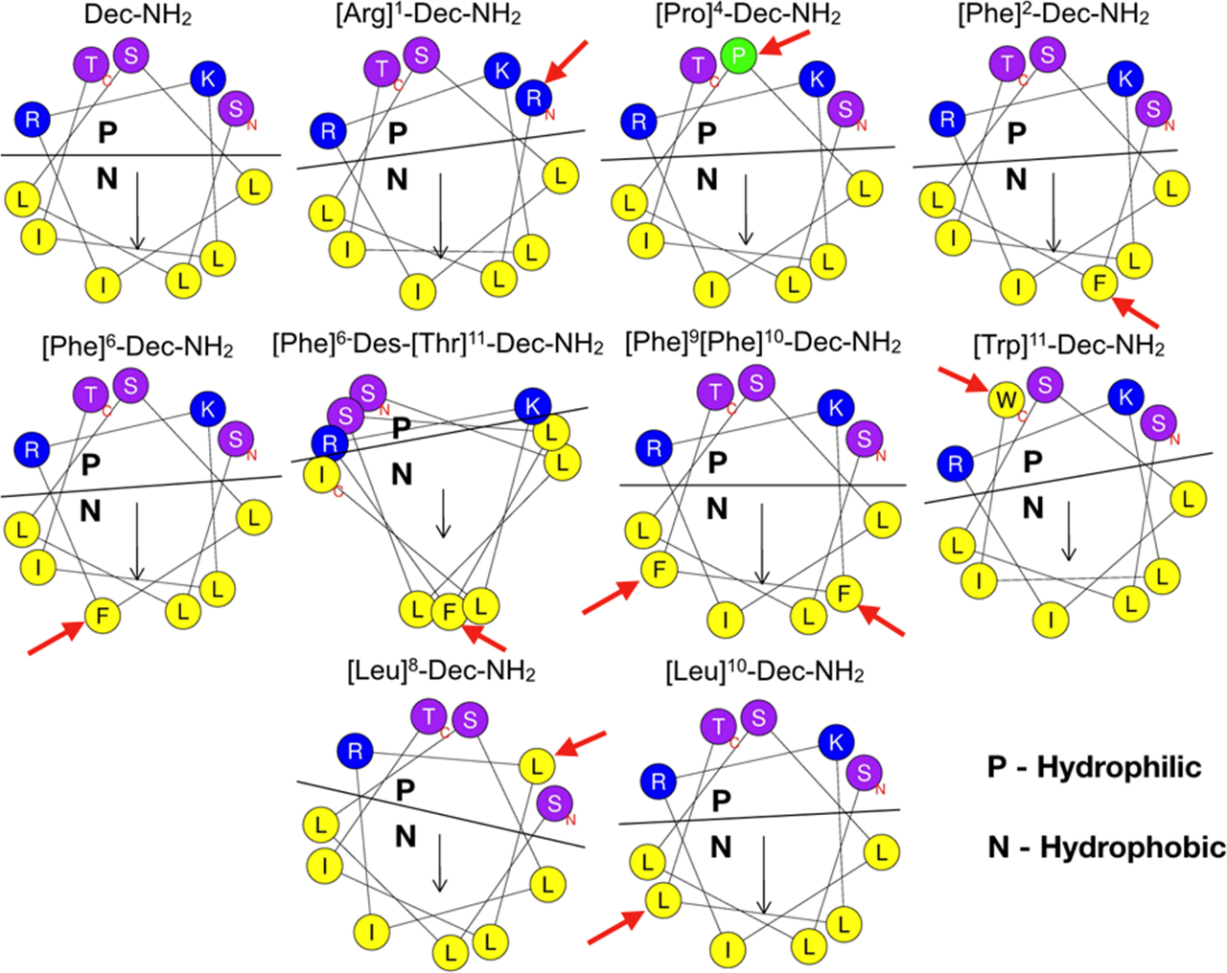
Helical wheel projections of Dec-NH_2_ and its analogs, where the yellow circles refer to the hydrophobic amino acid residues, the blue ones to the cationic charged residues, the purple circles to the polar uncharged residues and the green circle to a proline residue. The black line denotes hydrophilic and hydrophobic faces of the amphipathic structures. Red arrows show the mutation positions.

Dec-NH_2_ is a cationic α-helical antimicrobial and antiparasitic peptide [9–10,24] that is rich in Leu residues. We took into account these characteristics and designed two of the analogs proposed here using to a leucine zipper template, on which Leu residues were present in both ‘a’ and ‘d’ positions of the heptad sequence. This template design favors helical stabilization via Leu-side chain interactions [25,27] (Figure 1 – [Leu]^8^-Dec-NH_2_ and [Leu]^10^-Dec-NH_2_). The remaining Dec-NH_2_ derivatives were engineered by rationally introducing single and double substitution mutations (Figure 1). To introduce a net positive charge into the peptide sequences [28], we used Lys rather than Arg due to its superior flexibility, lower propensity in potentially toxic cell-penetrating peptides [29], and decreased hydrophobic side chain, which is associated with cytotoxicity [30]. Moreover, Lys residues are more frequent than Arg residues in naturally occurring wasp venom peptides [31]. Hydrophobicity was incorporated into the sequence via the substitution of residues from the wild-type sequence by Leu and Phe. Leu was chosen because a minimal amount of energy is required for it to adopt a helical structure [28], which favors antimicrobial activity, and it occurs at high frequency in wasp venom peptide sequences [31]. On the other hand, Phe was chosen because of its bulk and higher hydrophobicity values [30], making it possible to evaluate the effect of adding an aromatic residue to the hydrophobic face on structure and biological function. Additionally, unlike Trp, Phe residues are not major components of cell-penetrating peptides [32], which are typically cytotoxic, so we chose to synthesize a Trp-containing analog as well.

The changes in the designed analogs led to slight differences in specific physicochemical features (Table 1), such as hydrophobicity, hydrophobic moment, and net positive charge, characteristics that are known to be important for peptide–membrane interactions [10]. Some of these changes decreased the hemolytic activity against human red blood cells of Dec-NH_2_, reported by Konno et al. [24], and retained the antimicrobial activity described by Torres et al. [9–10] and the conformational tendency of peptides. In addition, the modifications led to an increased charge [9–10], an important feature that correlates with the improved therapeutic index of the Dec-NH_2_ derivatives and with the activity against microorganisms such as bacteria and fungi. Furthermore, Dec-NH_2_ and its analogs were hemolytic at concentrations above their MIC values for the different microorganisms studied [9–10].

**Table 1:** Theoretical physicochemical properties and hemolytic activity of decoralin and its synthetic analogs.^a^

peptide	sequence	H	μ_H_	q	MHC (μmol L^−1^)^b^	IC_50_ (μmol L^−1^)^c^

Dec-NH_2_	SLLSLIRKLIT-NH_2_	0.78	0.65	+3	1.56	12.5
[Pro]^4^-Dec-NH_2_	SLL**P**LIRKLIT-NH_2_	0.85	0.58	+3	12.50	25.0
[Arg]^1^-Dec-NH_2_	**R**LLSLIRKLIT-NH_2_	0.69	0.70	+4	25.00	50.0
[Phe]^2^-Dec-NH_2_	S**F**LSLIRKLIT-NH_2_	0.79	0.66	+3	3.12	50.0
[Phe]^6^-Dec-NH_2_	SLLSL**F**RKLIT-NH_2_	0.78	0.65	+3	3.12	>50
[Phe]^6^-Des[Thr]^11^-Dec-NH_2_	SLLSL**F**RKLI-NH_2_	0.83	0.39	+3	12.50	50.0
[Trp]^11^-Dec-NH_2_	SLLSLIRKLI**W**-NH_2_	0.96	0.49	+3	1.56	25.0
[Leu]^8^-Dec-NH_2_	SLLSLIR**L**LIT-NH_2_	1.03	0.48	+2	50.00	>50
[Leu]^10^-Dec-NH_2_	SLLSLIRKL**L**T-NH_2_	0.77	0.65	+3	25.00	12.5

^a^H (hydrophobicity), μ_H_ (hydrophobic moment), and q (charge) were calculated through heliquest freeware. MHC (maximal non-hemolytic concentration in μmol L^−1^). ^b^Maximal non-hemolytical concentration obtained by Torres et al. [9–10]. ^c^IC_50_ values against MCF-7 in 24 h.

### MTT cytotoxicity assays

**Figure 2:**
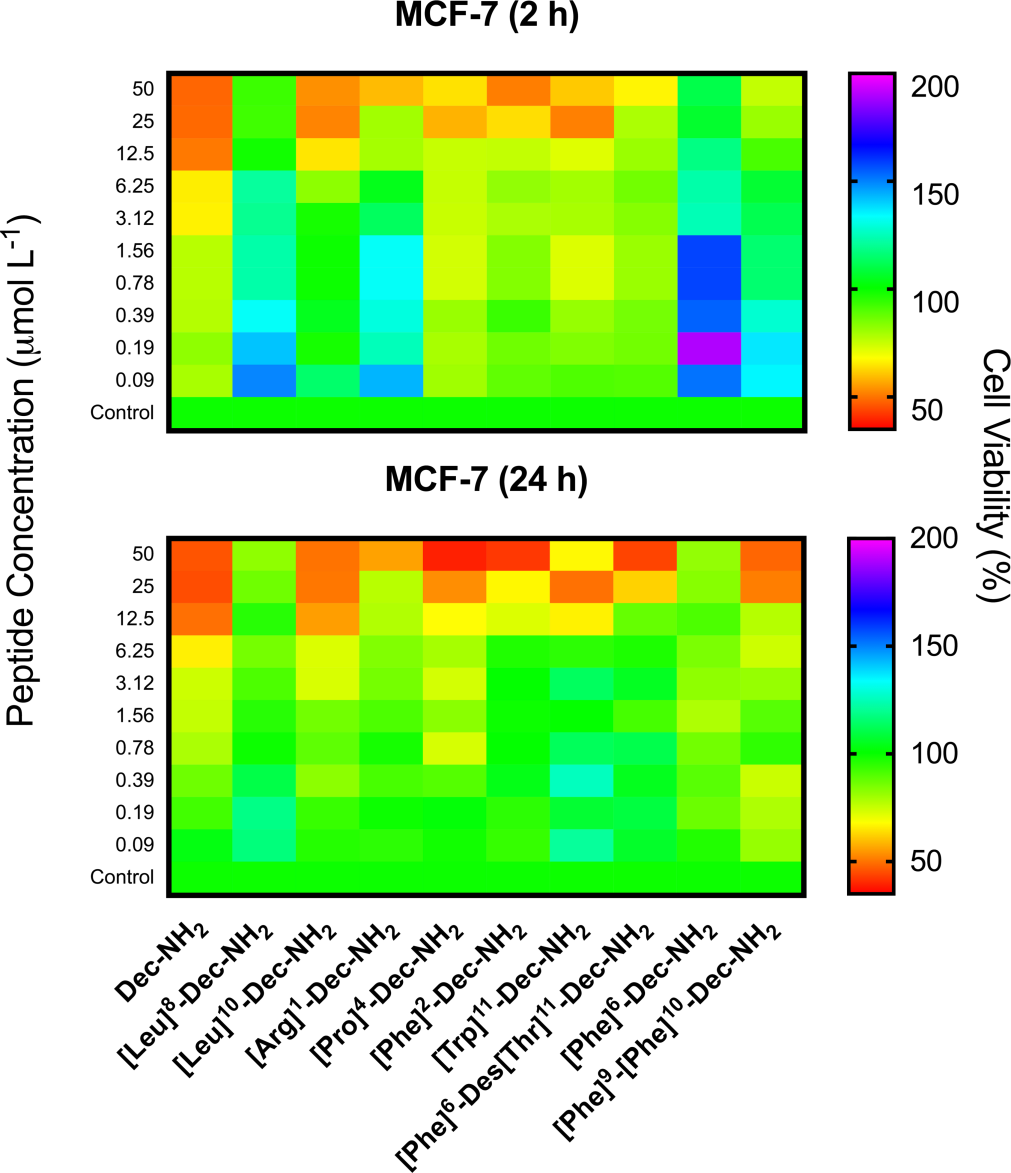
MTT assays using Dec-NH_2_ and its synthetic analogs after 2 and 24 h of exposure to MCF-7 cancer cells. Experiments were done in triplicate.

**Figure 3:**
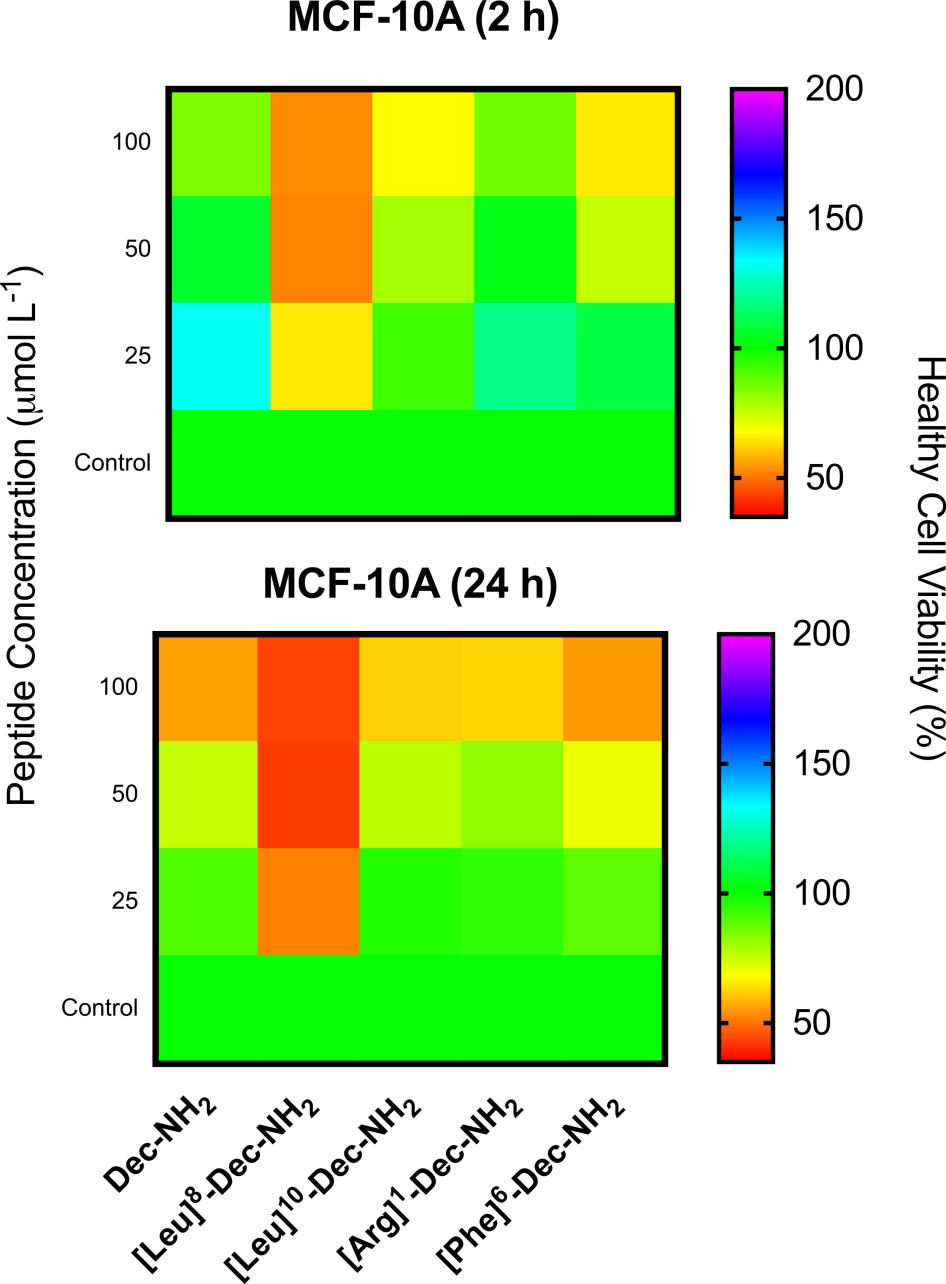
MTT assays evaluating the toxicity of Dec-NH_2_ and its derivatives towards MCF-10A normal cells after 2 and 24 h. Experiments were performed in triplicate.

MTT assays were performed to determine the toxicity of designer peptides against MCF-7 cancer cells and MCF-10A normal cells. MCF-10A cells were used as a control as they have the same genetic background as the MCF-7 cancerous cell line used here. Both cell types were treated with increasing concentrations of peptide for 2 and 24 h. ACPs are known to first interact with negatively charged membranes (i.e., cancer cell membranes) via electrostatic interactions, after which they tend to adopt helical conformations, which causes cell membrane permeabilization or even membrane disruption that may lead to necrosis [33]. These peptides may also be internalized into the cell, leading to the disruption of the mitochondrial membrane and causing apoptosis [33]. Torres et al. [9] described similar helical structure propensity and physicochemical properties for Dec-NH_2_ and [Leu]^10^-Dec-NH_2_. The main difference between these two peptides in terms of their biological function was the substantially lower hemolytic activity of the [Leu]^10^-Dec-NH_2_ analog, which yielded a higher therapeutic index. The antimicrobial activity of these peptides was nearly equivalent (10^−1^ μmol L^−1^). In contrast, the [Leu]^8^-Dec-NH_2_ analog presented a lower helical-structure tendency and almost no hemolytic activity vs human erythrocytes (Table 1), retained the antimicrobial activity of the WT, but was two orders of magnitude less active (10^1^ μmol L^−1^) than the [Leu]^10^-Dec-NH_2_ derivative. In Figure 2, it can be observed that, after 2 h, Dec-NH_2_ caused lysis of more than 50% of the cancer cells at 3.12 μmol L^−1^, and after 24 h, the LD_50_ value increased to 12.5 μmol L^−1^. [Leu]^10^-Dec-NH_2_ behaves similarly to the template molecule, achieving >50% of cancer cell lysis at 25 μmol L^−1^ after 2 h of exposure and at 12.5 μmol L^−1^ after 24 h. Their cytotoxicity levels were similar when tested against MCF-10A normal cells (Figure 3), showing no significant cytotoxicity even at higher concentrations (≈100 μmol L^−1^). On the other hand, [Leu]^8^-Dec-NH_2_ did not present significant activity against MCF-7 cells when compared to the negative control (Figure 2), and intriguingly was cytotoxic towards normal MCF-10A cells even at the lowest concentration tested (25 μmol L^−1^, Figure 3). This cytotoxicity is due to large differences in the values of the [Leu]^8^-Dec-NH_2_ physicochemical parameters that were analyzed, e.g., hydrophobicity related features, and lower net positive charge, compared to either [Leu]^10^-Dec-NH_2_ or the wild-type molecule, since the Leu substitution was made at the hydrophilic face of the amphipathic helical structure.

All the other analogs were designed by tuning some of the physicochemical features that contribute to peptide–membrane interactions in order to preserve the activity of the native sequence. Some of these changes decreased the hemolytic activity of Dec-NH_2_ towards human red blood cells reported by Konno et al. [24] and retained its antimicrobial activity. According to Torres et al. [10], the conformational tendency and increased charge are important contributors to improving the therapeutic index of Dec-NH_2_ and its derivatives against microorganisms such as bacteria and fungi. Furthermore, Dec-NH_2_ and its analogs were hemolytic at concentrations above their MIC vs the microorganisms tested. As observed in Figure 2, some of the peptides in this family showed promising results, causing substantial inhibition of cancer cell growth at a dose of ≈50 μmol L^−1^, e.g., Dec-NH_2_, [Pro]^4^-Dec-NH_2_, [Arg]^1^-Dec-NH_2_, [Phe]^2^-Dec-NH_2_ and [Phe]^6^-Des[Thr]^11^-Dec-NH_2_.

The analogs presented similar antitumor activity in growth inhibition assays with MCF-7 breast cancer cells. Dec-NH_2_, Trp- and Phe-substituted analogs were described as the most hemolytic peptides of their family [23]. Treatment with peptide [Arg]^1^-Dec-NH_2_ led to significant decreased cell viability 2 h post-exposure (Figure 2) but was not as effective vs MCF-7 cells as its parent peptide. This peptide was selected for cytotoxicity assays against normal cells because it was not as hemolytic as the wild-type and the other derivatives evaluated (Table 1) and presented higher antimicrobial activity when compared to the other analogs that also exhibited anticancer activity, such as [Pro]^4^-Dec-NH_2_ and [Phe]^6^-Des[Thr]^11^-Dec-NH_2_ (Figure 2).

We also observed noticeable differences among the Phe-substituted peptides. For instance, [Phe]^2^-Dec-NH_2_ and [Phe]^6^-Des[Thr]^11^-Dec-NH_2_ inhibited cell viability the most, at 50 μmol L^−1^ after 2 h (Figure 2). On the other hand, [Phe]^6^-Dec-NH_2_ did not show significant inhibition after 2 h and [Phe]^9^-[Phe]^10^-Dec-NH_2_ did only show significant inhibition after 24 h (Figure 2). [Phe]^6^-Des[Thr]^11^-Dec-NH_2_ did not present helical tendencies, as analyzed by Torres et al. [10], and was not as hemolytic as the other Phe-substituted analogs (Table 1).

[Pro]^4^-Dec-NH_2_ was described as an unstructured peptide even in helical promoter media [34–35] by Torres et al. [10] and was relatively hemolytic (Table 1) [10], but it decreased MCF-7 cancer cell viability more substantially after 24 h than after 2 h (Figure 2). [Trp]^11^-Dec-NH_2_, which had the highest hemolytic activity among the peptides of the Dec-NH_2_ family (Table 1), significantly inhibited viability of MCF-7 cells at 25 μmol L^−1^ after 2 h (Figure 2).

### Cell death assays

**Figure 4:**
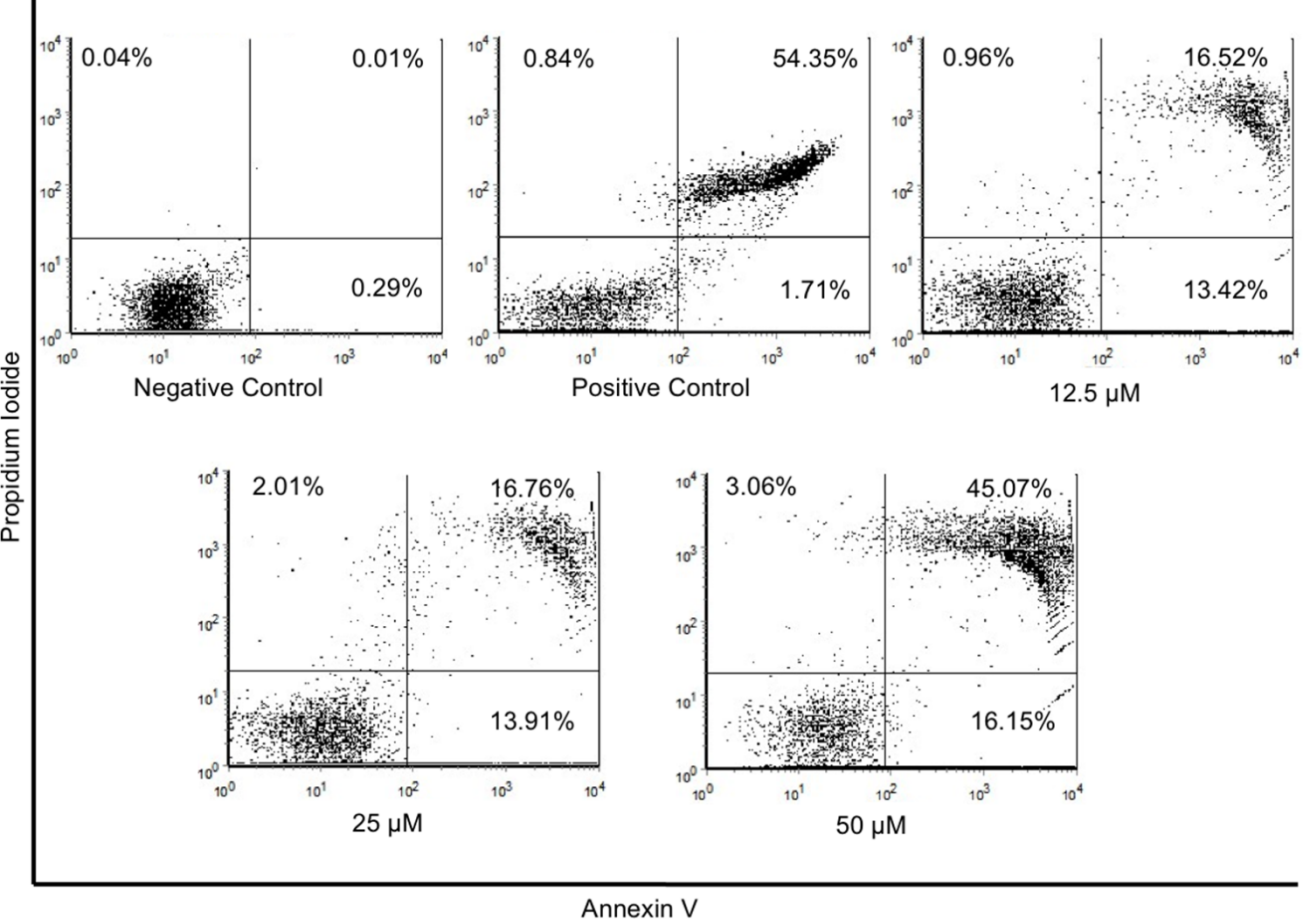
Cell death analysis using flow cytometry. Dot plot graphs from left to right, show cells treated with: (negative control) MCF-7 cells labeled with Annexin/PI, and (positive control) 2.0 μmol L^−1^ staurosporine labeled Annexin V-FITC and PI. Dot plot of MCF-7 cells after exposure to 12.5, 25 or 50 μmol L^−1^ of Dec-NH_2_ for 24 h, and flow cytometry analysis with Annexin V-FITC versus PI. The divisions of the plots distinguish necrotic cells (Annexin V+/PI+, right upper quadrant) from apoptotic cells (Annexin V+/PI−, right lower quadrant).

Flow cytometry experiments were performed in an attempt to obtain insight into the mechanism of peptide-mediated death of cancer cells. For these proof-of-concept assays, we focused on WT peptide Dec-NH_2_. We utilized Annexin V labeling FITC (X axis) and propidium iodide (PI, Y axis). Under these conditions, (Annexin V+/PI+, right upper quadrant) were interpreted as necrotic cells and (Annexin V+/PI−, right lower quadrant) as apoptotic cells (Figure 4). As a positive control, we treated cells for 1 h with a solution of 2.0 μmol L^−1^ staurosporine (Figure 4).

Cells treated with 12.5 and 25 μmol L^−1^ of Dec-NH_2_ showed approximately 16% of cells in the necrotic stage and around 14% of cells in the apoptotic stage after 24 h of incubation. However, the percentage of necrotic cells increased approximately three times (to 45%), when the concentration of Dec-NH_2_ was increased to 50 μmol L^−1^. This is consistent with the MTT assay results obtained with the same peptide (Figure 2), indicating that Dec-NH_2_ triggers membrane disruption thus leading to cell death and necrosis of cancer cells.

### AFM measurements

**Figure 5:**
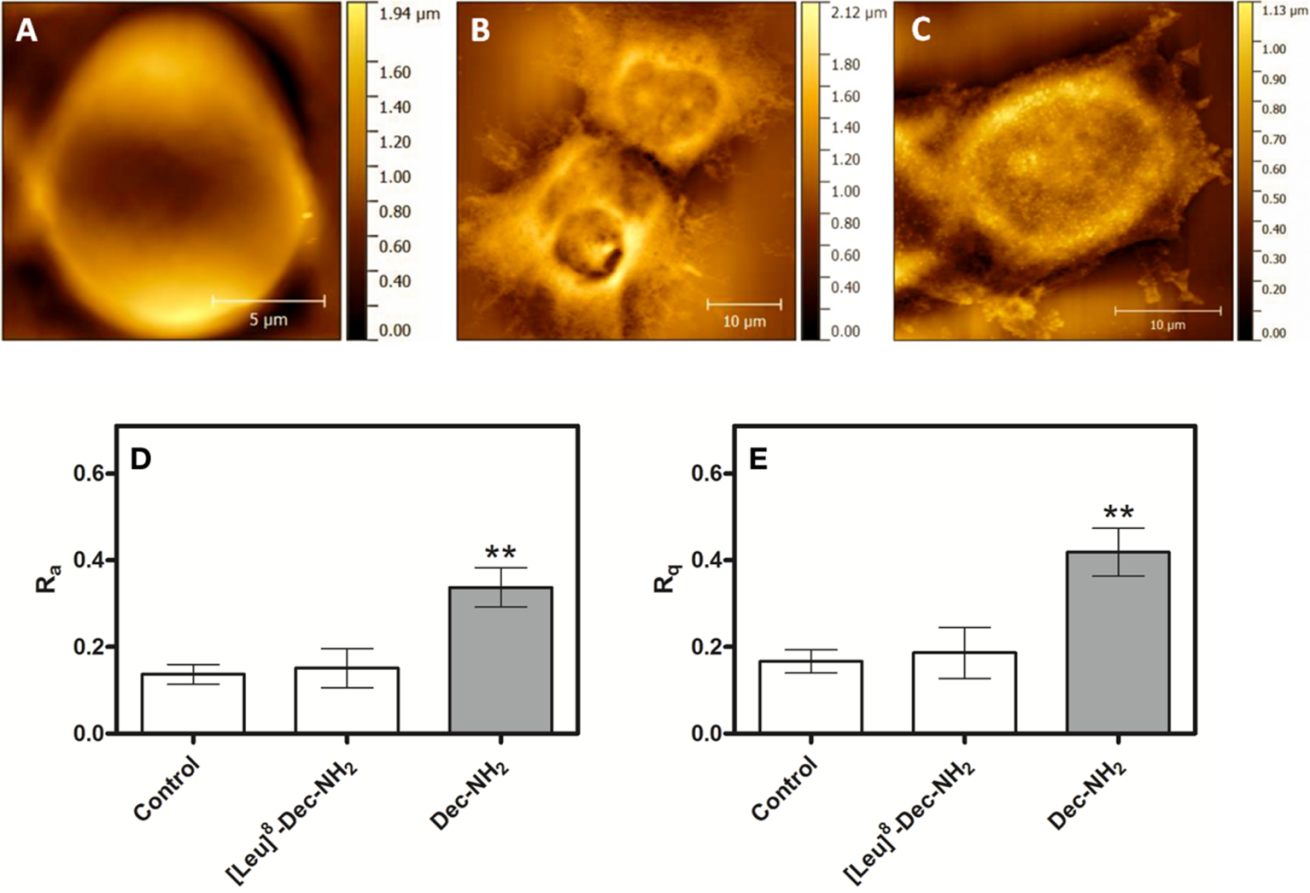
Topological images of untreated MCF-7 cells (A) and cells treated for 24 h with 50 μmol L^−1^ of Dec-NH_2_ (B) or 50 μmol L^−1^ of [Leu]^8^-Dec-NH_2_ (C). Roughness values of membranes of untreated MCF-7 cells and of those cells treated with peptides. (D) Data represent the mean values of the surface relative to the center plane of measurements ± standard deviations (*n* = 5). (E) The root mean square of the values and the standard deviation of the area were analyzed. More than 5 points were measured per sample. Significant differences between peptide-treated and untreated cells are given by *p* > 0.05 (*).

AFM was used to quantify the cellular structure (i.e., membrane roughness) of MCF-7 cells upon peptide treatment in order to determine whether cell topology was disturbed, as changes in topology would provide further insight into the mechanism of action of our lead peptides. Cantilevers in contact mode were used to obtain the topographic images from different areas of treated and untreated cell samples [36], and representative results are shown in Figure 5A–C. Peptides Dec-NH_2_ and [Leu]^8^-Dec-NH_2_ were chosen as control peptides as they were the most and least potent, respectively, vs MCF-7 cells as determined by MTT assays (Figure 2).

Exposure of MCF-7 cells to positive control peptide Dec-NH_2_ for 24 h increased cancer cell membrane roughness by approximately 100% compared with cells from the untreated control group (Figure 5D,E). Conversely, treatment with negative control peptide [Leu]^8^-Dec-NH_2_ did not significantly change membrane roughness (Figure 5D,E). Our data indicates that peptide treatment leading to membrane disruption and subsequent cell death is associated with changes in the membrane of cancer cells, specifically, greater roughness.

The AFM results are in line with the activity of the peptides obtained in MTT assays, which highlights the importance of certain physicochemical properties for the bioactivity of these two peptides, in line with previous work by Torres et al. [9]. Currently, there is no consensus on how the biophysical properties of peptides influence their antimicrobial and antitumoral activities. However, in the specific case of Dec-NH_2_ and its analogs, helical propensity, having higher hydrophobicity, hydrophobic momentum, and displaying a net positive charge appeared to correlate with improved antitumoral activity. These results add to our current understanding of the structure–activity relationships of ACPs and may lead to novel insights about the innate immune system and to new peptide-based anticancer chemotherapies.

## Conclusion

Current cancer treatments are associated with numerous harmful side effects, which warrants the discovery of novel forms of treatment. ACPs have been proposed as novel anticancer therapies because of their potential for selectively targeting cancer cells without harm to normal counterparts [37–39].

Membrane phospholipids confer permeability to the cell and regulate the flux of metabolites between the extracellular environment and the intracellular content [40]. The membrane of cancer cells is typically negatively charged due to a higher expression of anionic molecules such as phosphatidylserines, and negatively charged glycoproteins and glycosaminoglycans [22–23]. Here, we devised a strategy to exploit the negatively charged environment of cancer cells by targeting it with cationic peptides. This strategy is based on the electrostatic interaction of the peptides, through their cationic residues, with the anionic phospholipids present in the membrane [39–40]. The peptides accumulate in the membrane, leading to perturbation of membrane integrity and subsequent cell death [40–42].

We present results obtained with the naturally occurring peptide Dec-NH_2_ derived from wasp venom and with its mutant analogs containing single and double substitutions. These peptides, which had been previously shown to display antimicrobial properties [9–10], exhibited anticancer activity against MCF-7 breast cancer cells at concentrations ranging from 12.5 to 50 μmol L^−1^ (Figure 2). The lead anticancer peptides were tested against healthy breast tissue from the same cell line background (MCF-10A) and were shown to selectively target cancer cells. The peptides’ selectivity observed towards cancer cells versus normal cells is likely due to the acidic microenvironment that accompanies cancer cells, and the increased net negative charge of cancer cells versus normal cells, which display a net neutral charge [12,20].

The mechanism of peptide-mediated cell death was further analyzed using flow cytometry for the WT peptide (Dec-NH_2_). Peptide treatment led to necrotic death of cancer cells. Additional AFM experiments revealed that the roughness of the cancer cell membrane increased significantly when treated with this peptide, when compared with untreated cells or cells treated with the negative control peptide [Leu]^8^-Dec-NH_2_. These results indicate that peptide treatment alters the ultrastructure of the cancer cell membrane, an alteration that is apparently part of the observed anticancer activity.

The biophysical features of peptides play an important role in peptide–membrane interactions. Here, we designed peptide variants derived from Dec-NH_2_, taking into account key physicochemical properties of ACPs, such as hydrophobicity, amphipathicity, and positive net charge. Our results show that significant changes in amphipathicity, net charge, and hydrophobicity led to decreased activity against MCF-7 cancer cells ([Leu]^8^-Dec-NH_2_ and [Phe]^6^-Dec-NH_2_ analogs) and, in some cases, to unwanted effects, such as increased cytotoxicity against normal MCF-10A cells (e.g., [Leu]^8^-Dec-NH_2_). In addition, we identified [Leu]^10^-Dec-NH_2_ as an excellent candidate with which to pursue the use of ACPs for eventual clinical development, as it displayed reduced hemolytic activity than Dec-NH_2_ and exhibited selective killing of cancer cells. The ACPs described here represent excellent scaffolds for the generation of potent, non-toxic, and selective anticancer agents.

## Experimental

### Peptide synthesis, purification and analysis

Peptides were synthesized by solid-phase peptide synthesis on Rink Amide resin, with a substitution degree of 0.52 mmol g^−1^ on a 0.1 mmol scale, using the Fmoc strategy on a peptide synthesizer (PS3 – Protein Technologies) as described by Torres et al. [9–10].

Dry-protected peptidyl-resin was exposed to TFA/anisole/water (95:2.5:2.5, v/v/v) for 2 h at room temperature. The crude deprotected peptides were precipitated with anhydrous diethyl ether, filtered from the ether-soluble products, extracted from the resin with 60% ACN (acetonitrile) in water and lyophilized.

The crude lyophilized peptides were then purified by preparative reversed-phase high-performance liquid chromatography (RP-HPLC) in 0.1% TFA/90% ACN in water (A/B) on a Delta Prep 600 (Waters Associates). Briefly, the peptides were loaded onto a Phenomenex C_18_ (21.2 mm × 250 mm, 15 µm particles, 300 Å pores) column at a flow rate of 10.0 mL min^−1^ and eluted using a linear gradient (0.33% B/min slope), with detection at 220 nm. Selected fractions containing the purified peptides were pooled and lyophilized. Purified peptides were characterized by liquid-chromatography electrospray-ionization mass spectrometry (LC/ESIMS).

LC/ESIMS data were obtained on a Model 6130 Infinity mass spectrometer coupled to a Model 1260 HPLC system (Agilent), using a Phenomenex Gemini C_18_ column (2.0 mm × 150 mm, 3.0 μm particles, 110 Å pores). Solvent A was 0.1% TFA in water, and solvent B was 90% ACN in solvent A. Elution with a 5–95% B gradient was performed over 20 min, 0.2 mL min^−1^ flow and peptides were detected at 220 nm. Mass measurements were performed in a positive mode with the following conditions: mass range between 100 to 2500 *m/z*, ion energy of 5.0 V, nitrogen gas flow of 12 L min^−1^, solvent heater of 250 °C, multiplier of 1.0, capillary of 3.0 kV and cone voltage of 35 V.

### Cell culture and treatment

MCF-7 cells (ATCC) were maintained in RPMI 1640 medium supplemented with 10% heat inactivated FBS and 100 μg mL^−1^ penicillin/10 μg mL^−1^ streptomycin. One day before the assays, the cells were plated in 96-well microtiter plates with a density of 2.0 × 10^4^ cells/well at 37 °C and 5% CO_2_. On the next day, cells were treated with peptides serial dilutions (0.09–50 μmol L^–1^), incubated in individual microtiter plates for 2 and 24 h and MTT assays were performed after treatment. Human breast epithelial cells MCF-10A (ATCC) were maintained in a mixture of Dulbecco’s Modified Eagle’s Medium and Ham’s F12 nutrient mixture supplemented with 5% inactivated horse serum, 10 μg mL^−1^ insulin, 0.02 μg mL^−1^ human epidermal growth factor, 0.5 μg mL^−1^ hydrocortisone, 0.10 μg mL^−1^ choleric toxin, 100 U mL^−1^ penicillin, and 100 μg mL^−1^ streptomycin. The cells were preincubated for 24 h, plated in 96-well microtiter plates with a density of 2.0 × 10^4^ cells/well at 37 °C and 5% CO_2_. On the next day, cells were treated with peptides serial dilutions (25 to 100 μmol L^−1^), incubated in individual microtiter plates for 4 and 24 h and MTT assay was performed after treatment. Experiments were performed in triplicate.

### MTT assay

Briefly, MTT (Sigma-Aldrich) was dissolved in water and filtered to make up a 5 μg mL^−1^ solution. 30 μL of this solution were added to all the wells which already contained peptide-treated cells and kept at 37 °C for 45 minutes. Subsequently, the solution was discarded and replaced with 150 μL/well of DMSO and followed by gentle shaking for 15 minutes. Finally, the microplates were read on an ELISA reader at 570 nm. Experiments were performed in triplicate.

### Cell death assay

The percentage of cells undergoing apoptosis and necrosis was determined by Annexin V/propidium Iodide staining using the ApopNexin^TM^ FITC Apoptosis Detection Kit (Millipore) in a flow cytometer (BD Facs Canto II - BD). MCF-7 cells were seeded in 6-well plates and treated for 24 h with 12.5, 25 or 50 μmol L^−1^ Dec-NH_2_ solution and 2.0 μmol L^−1^ staurosporine in water (positive control). The apoptosis assay was performed according to Matias et al. [36].

### AFM measurements

The AFM imaging of MCF-7 cells untreated (control) and treated with peptide (50 μmol L^−1^ solutions of Dec-NH_2_ and [Leu]^8^-Dec-NH_2_, which presented low activity when compared to other analogs and was used here as a treated control) was performed using an Agilent Technologies 5500 AFM/SPM microscope that was in contact mode and a Nanosensors™ PPP-CONT probe (NanoSensors; PPP-Cont-20, PointProbe-Plus Silicon-SPM-Sensor). The material properties and dimensions of the AFM tips used in this experiment were as follows: resonance frequency of 6–21 kHz, force constant of 0.02–0.77 N m^−1^, cantilever length of 450 ± 10 μm, cantilever width of 50 ± 7.5 μm, cantilever thickness of 2 ± 1 μm, tip height of 10–15 μm and resistivity of 0.01–0.02 Ω cm. The assays were performed in triplicate; image processing and roughness determinations were performed with the aid of the Gwyddion software (http://gwyddion.net/download.php). In order to compare the cell surface, we used two roughness parameters, the mean roughness (*R*_a_) and the mean square of Z data (*R*_q_), where *N* is the difference between the highest and the lowest points in the analyzed area. These parameters should not be considered as absolute roughness values because they strictly depend on the tip used in the assays.

*R*_a_ is the mean value of the surface relative to the center plane of the measurements. This plane is defined by where the volumes enclosed by the image above and below are equal and it is given by Equation 1:

[1]
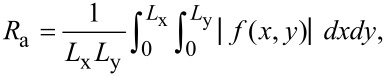


where *f*(*x,y*) is the surface relative to the center plane and, *L*_x_ and *L*_y_ are the surface dimensions.

The root mean square of the *Z* values *R*_q_ is the standard deviation of the *N* values in the area analyzed and is given by Equation 2:

[2]
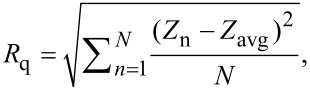


where *Z*_avg_ is the average of the *Z* values in the given area, *Z*_n_ is the current value, and *N* is the number of points in this area [43].
